# Parathyroid autotransplantation: a phase-based framework for surgical decision-making

**DOI:** 10.3389/fendo.2026.1863354

**Published:** 2026-07-09

**Authors:** Hanyun Tu, Yuhan Jiang, Tianyuchen Jiang, Yi Yang, Anping Su

**Affiliations:** Department of General Surgery, West China Hospital of Sichuan University, Chengdu, China

**Keywords:** autograft, autotransplantation, parathyroid, surgery, transplantation

## Abstract

Parathyroid autotransplantation (PAT), a technique in which parathyroid tissue is excised from its original site and transplanted to an alternative site within the same individual, serves as a pivotal preventive and therapeutic strategy for hypoparathyroidism. Its success is not guaranteed by a single intervention but is determined by a series of crucial decisions that directly influence autograft survival and function, such as the physiological or pathological state of the parathyroid gland, revascularization, and the quality of the surgical process. This review comprehensively delineates the PAT procedure as a continuous, phase-based process, including intraoperative parathyroid identification and evaluation techniques; determining whether to perform PAT and the quantity of autotransplanted tissue; selecting immediate or delayed PAT; processing parathyroid autografts; choosing the PAT site; and methods for evaluating autograft function. By synthesizing the latest evidence for each of these critical phases, this novel, phase-based perspective not only provides a structured framework to guide surgical decision-making but also highlights the research and development potential of PAT, with the ultimate goal of optimizing patient outcomes.

## 1 Introduction

The parathyroid glands are pivotal regulators of systemic calcium and phosphate homeostasis through the secretion of parathyroid hormone (PTH). This hormonal control is critical for vital physiological processes, including neuromuscular excitability, bone metabolism, and cardiovascular function. However, the glands are susceptible to inadvertent injury, devascularization, or intentional removal during neck surgeries, potentially causing hypoparathyroidism.

Parathyroid autotransplantation (PAT) is a surgical procedure that involves transplanting excised parathyroid tissue to an alternative site within the same individual, primarily to prevent or treat hypoparathyroidism. Its success relies on the regenerative capacity of parathyroid cells, which can revascularize and resume PTH secretion at a new site with adequate blood supply. Due to its established efficacy in preventing and treating hypoparathyroidism, PAT is widely applied across a broad spectrum of surgical indications. Its application extends from thyroidectomy to the surgical management of primary hyperparathyroidism (PHPT), secondary hyperparathyroidism (SHPT), and hyperparathyroidism (HPT) associated with genetic syndromes such as multiple endocrine neoplasia (MEN).

**Figure 1:**
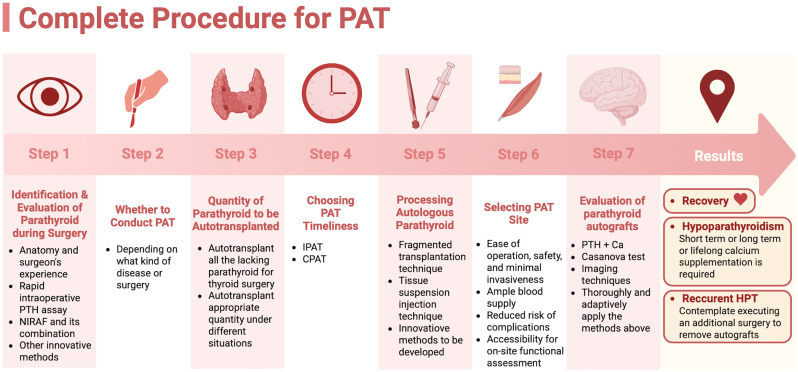
Complete phase-based procedure for PAT. PAT, parathyroid autotransplantation; PTH, parathyroid hormone; NIRAF, near-infrared light autofluorescence; IPAT, immediate parathyroid autotransplantation; CPAT, cryopreserved parathyroid autotransplantation; HPT, hyperparathyroidism.

However, the implementation of PAT varies considerably across different indications. This variation stems from factors such as the underlying disease pathology, the required extent of surgery, and individual patient characteristics. Consequently, a personalized surgical strategy is required, involving critical decisions regarding intraoperative parathyroid identification and evaluation, determining whether and how much tissue to transplant, selecting PAT timing, processing the tissue, choosing the PAT site, and evaluating postoperative autograft function (**Figure 1**). Understanding these phases is paramount to optimizing graft survival and patient outcomes. This review consolidates a novel phase-based overview of these critical aspects into an integrated framework, synthesizing current evidence to guide clinical practice.

## 2 Methods

This manuscript was prepared as a narrative review to provide a structured, evidence-based guide for surgical decision-making, offering a comprehensive overview that can help optimize PAT outcomes. The review was not designed to exhaustively identify all eligible studies or formally rate study quality, as would be expected in a systematic review.

A comprehensive literature search was conducted using the electronic databases PubMed, Web of Science, Embase, and Cochrane, from database inception through January 2026. Search strategies combined key terms and MeSH terms, including “parathyroid gland”, “autotransplantation”, “thyroidectomy”, “primary hyperparathyroidism”, “secondary hyperparathyroidism”, and “multiple endocrine neoplasia”. The full search strategy is provided in **Supplementary Table 1**. To enhance topic coverage within a narrative framework, reference lists of relevant review articles and key primary studies were also screened to identify additional publications not captured by the initial keyword combinations.

Since the objective was conceptual synthesis rather than systematic enumeration, study selection was based on relevance to the phase−based PAT framework. Exclusion criteria included (1): studies with fewer than five cases (2); non−English publications (3); publications that were not original research articles, case studies, or review articles; and (4) studies in which neither PAT techniques (e.g., intraoperative parathyroid identification/evaluation, PAT versus *in situ* preservation, immediate vs. delayed PAT, tissue processing methods, or transplantation sites) nor outcome measures were clearly described in the Methods or Results sections. Given the narrative design, no PRISMA flow diagram was produced, and no formal risk−of−bias tool was applied. Instead, divergent findings were addressed qualitatively by considering study design features such as baseline patient characteristics, surgical indications, PAT protocols, outcome assessment, and related methodological factors.

This review is based on previously published studies and does not involve new data collection from human participants or animals.

## 3 Parathyroid identification and evaluation techniques during surgery

Accurate intraoperative identification and evaluation of parathyroid glands are essential (**Table 1**). The most convenient approach is visual recognition (which can be assisted by magnifying glasses), based on anatomical knowledge and gland characteristics (typically 3 x 3 x 3mm, yellow-brown, and located on the thyroid posterolateral capsule) (1, 2). The upper parathyroids are often located at the junction of the inferior thyroid artery and recurrent laryngeal nerve, while the lower glands are typically found at the thyroid inferior pole or in the thyrothymic ligament (3). Ectopic parathyroids occur in 23.8% of cases, predominantly involving the left lower gland in the thymus or superior mediastinum (4). Gland viability can be assessed intraoperatively through color changes and capsule bleeding using an incision test (5, 6). Nevertheless, a normal-looking gland may not be functional due to potential vascular thrombosis or edema, underscoring the importance of surgeons’ accumulated experience and judgment.

**Table 1:** Intraoperative parathyroid identification, evaluation methods, and postoperative autograft function assessment methods.

Phase	Technical category	Specific technique	Core principle	Key indicators	Main advantages	Limitations and clinical considerations
Intraoperative parathyroid identification and viability assessment	Conventional and basic methods	Visual identification and anatomy	Based on typical gland appearance (yellow-brown, ovoid, ~3×3×3 mm) and common anatomical locations.	Surgeon’s subjective judgment. Superior glands often at junction of inferior thyroid artery and recurrent laryngeal nerve; inferior glands near thyroid lower pole or in thyrothymic ligament.	Convenient, quick, no additional cost, fundamental to all procedures, may be aided by magnifying glasses.	Highly experience-dependent; low recognition rate for ectopic or small glands.
		Incision test	Sharp tenotomy scissors are used to take an incisional biopsy of each suspicious parathyroid.	The evidence of active bleeding suggests adequate blood supply and viability.	Direct, rapid *in-situ* identification of glands.	Invasive; may damage the vascular pedicle.
	Biochemical rapid assays	Intraoperative rapid PTH aassay	Monitors decrease in peripheral blood iPTH concentration after thyroid resection.	iPTH <10 ng/L 10–20 minutes post-resection predicts hypocalcemia risk and can guide PAT decision.	Objective, quantitative, dynamically reflects overall residual parathyroid function.	Requires lab support, time delay; reflects global function not individual glands.
		ECLIA	Conduct suspicious tissue FNA. The PTH concentration in the washout fluid is quantitatively measured using ECLIA.	PTH concentration is extremely high in parathyroid tissue vs. non-parathyroid tissue (P<0.0001).	Provides a highly reliable, quantitative measurement.	Invasive sampling; Requires ECLIA instrument; processing time (15min) still requires waiting.
		ICGT	The PTH in the sample forms a complex with gold-labeled antibodies, which is captured at the test zone, producing a colored band whose intensity is proportional to PTH concentration.	Distinct PTH thresholds for identification: ~63.99 pg/mL for FNA samples and ~136.30 pg/mL for TBH. Provides a rapid, visual readout.	Extremely fast, portable, and user-friendly. Superior diagnostic accuracy (98.6%) compared to direct visual inspection (74.1%). Enables both *in situ* FNA and *in vitro* TBH identification.	Provides semi-quantitative results. FNA method has a lower sensitivity, potentially due to sampling error. TBH requires tissue resection, which is destructive to the sample.
	NIRAF and its combinations	NIRAF	Intrinsic fluorophores in parathyroid cells emit ~820 nm fluorescence when excited by ~800 nm light, ~11× brighter than adjacent tissues.	Detection device shows fluorescence signal intensity higher than background in gland areas.	Label-free, real-time, high sensitivity, no contrast agent needed.	Provides identification only, cannot assess gland viability.
		ICG	Intravenous injection of ICG dye. The dye binds to plasma lipoproteins and fluoresces under near-infrared light, highlighting vascularized tissues.	ICG fluorescence visualizes the parathyroid glands approximately 203 seconds post-injection, lasting about 20.8 minutes.	Real-time, intuitive assessment of gland vascularization, high detection rate. Significantly reduces incidental parathyroidectomy.	The ICG uptake in the thyroid limits the ability to distinguish parathyroids, with a 6% detection rate. Requires IV injection, with potential for allergic reactions. Contains iodine, caution needed in allergic/renal impairment patients.
		LSCI	Blood vessels generate speckle contrast under laser beam irradiation.	High blood flow areas appear as low speckle contrast, while low blood flow areas exhibit high speckle contrast.	Label-free, non-contact, real-time quantitative assessment of blood flow with high accuracy. Combine with NIRAF to make ParaSPAI.	LSCI: Susceptible to motion artifacts from vibration, respiration, and surgical traction. ParaSPAI device is complex and expensive.
	Emerging techniques	BIS	Measures tissue impedance spectrum across frequencies, reflecting differences in cellular structure and composition.	Can distinguish parathyroid from thyroid, lymph node, and adipose tissue.	Provides fast objective discrimination based on tissue electrical properties with high accuracy, sensitivity and specificity.	Requires larger clinical validation. Performance may vary with tissue temperature and probe contact.
		HSI	Utilizes light spectrum analysis to characterize tissues.	Mean accuracy: 68 ± 23%; parathyroid sensitivity: 65 ± 17%, specificity: 94 ± 6%; thyroid sensitivity: 75 ± 29%, specificity: 96 ± 3%.	Contactless, real-time visualization, quick multi-tissue discrimination.	Low nerve detection sensitivity; performance varies with pathology; small sample size.
		FLIm	Using ultraviolet induced fluorescence decay time to differentiate tissue types.	100% sensitivity, 93% specificity, 97% accuracy.	Not affected by ambient light; no need for external contrast agents; rapid signal integration without calibration; enables real-time mapping with overlay on surgical view.	Limited to point measurements in this study; occasional false positives with lymphoid tissue due to signal variation; small sample size.
		OCT Imaging	Utilizes low-coherent, near-infrared light and interferometry to generate high-resolution, cross-sectional images of tissue microstructure in real-time.	High accuracy in identifying thyroid tissues: normal (82.4%), nodular (84.3%), cancerous (100%). Lower accuracy for parathyroid (52.3%), lymph node (32.4%), and fat (55.9%).	Real-time, non-invasive “optical biopsy”; high resolution (~10 μm) for microstructural details; portable and easy to operate.	Limited penetration depth (2 mm), restricting evaluation to superficial layers; potential false readings if tissue is surrounded by fat.
		DOCI	Integrates dynamic contrast with optical imaging to evaluate tissue function and structure throughout time.	100% Sensitivity, 98.8% specificity, capability to tell normal parathyroid from diseased ones and adjacent healthy thyroid.	Non-invasive, label-free, and safe.	Currently limited to ex vivo tissue studies, requires development for *in vivo* use.
Postoperative autograft function assessment	Biochemical gold standard	Serum PTH & Calcium Monitoring	Functional transplanted tissue should autonomously secrete sufficient PTH to maintain calcium homeostasis.	After stopping supplements, serum iPTH and calcium remain normal. Permanent failure: more than one-year post-op requiring meds with subnormal or undetectable iPTH.	Gold standard for determining physiological function of autografts.	Must be assessed after stopping calcium/active vitamin D, otherwise function overestimated.
	Functional localization tests	Casanova Test	Cuff occlusion of transplanted forearm blood flow, observing the change of iPTH.	iPTH decrease >46% after occlusion suggests PTH mainly from forearm graft (positive).	Classic functional method to determine PTH source.	Causes patient discomfort, ischemic risks.
		Modified Casanova Test	Surviving functional graft secretes PTH entering venous return of transplanted side first, creating concentration gradient.	Simultaneous bilateral forearm venous blood PTH: transplanted/non-transplanted side ratio >1.5 indicates normal graft function.	Minimally invasive, highly specific, better patient tolerance.	Sampling site should be near graft area; only for forearm grafts.
	Imaging assessment	High-Frequency Color Doppler Ultrasound	Uses high-frequency sound waves to visualize microstructure, color Doppler shows internal/peripheral blood flow.	Small nodular hypoechoic foci in target muscle/subcutis with blood flow signals inside or at the peripheries.	Convenient, radiation-free.	Limited sensitivity.
		^99^mTc-MIBI SPECT/CT	^99^mTc-MIBI as a lipophilic cation, can be captured and retained in large quantities by mitochondria, with hyperparathyroid cells exhibiting increased uptake due to their more abundant and active mitochondria, thus remaining in the cell for a longer period of time.	Focal radiotracer uptake at transplant site on SPECT/CT fusion images.	Combines metabolic function & anatomical localization.	Moderate sensitivity (57%).
		^18^F-Choline PET/CT	Choline incorporated into membrane phospholipids; proliferating parathyroid cells show increased uptake. ^18^F half-life ~110 min, ¹¹C half-life ~20 min.	Focal radioactive uptake at transplant site distinct from background.	High sensitivity (90.3%), especially valuable after negative/equivocal ^99^mTc-MIBI.	Higher cost.
		¹¹C-Choline PET/CT			Very high sensitivity (96.3%), especially valuable after negative/equivocal ^99^mTc-MIBI or ^18^F-Choline.	Expensive.
		MRI	Strong magnetic fields and radio frequency waves can cause hydrogen protons in human water molecules resonate and absorb energy. Different organs recover hydrogen protons at different speeds.	Well-functioning grafts show T2WI iso-/slightly hyperintense signals, T1WI+c pronounced uniform enhancement post-contrast.	No radiation, points to a healthy tissue with intact cellular structure, normal water content, and abundant blood supply, which possesses the capacity to secrete PTH biologically.	Indirect evidence for functional activity.
		CT	Using X-rays to rotate and scan certain layers of the body, the detector collects attenuated signals that penetrate the body, and the computer reconstructs the cross-sectional image.	Shows presence of soft tissue nodules at transplant site.	High spatial resolution, precise anatomical localization, assist preoperative planning.	Provides limited functional information; radiation exposure.

PTH, parathyroid hormone; iPTH, intact parathyroid hormone; ECLIA, electrochemiluminescence immunoassay; FNA, fine-needle aspiration; ICGT, immunological colloidal gold technique; TBH, tissue block homogenate; NIRAF, near-infrared light autofluorescence; ICG, indocyanine green; LSCI, laser speckle contrast imaging; ParaSPAI, parathyroid speckle and autofluorescence imager; BIS, bioelectrical impedance spectroscopy; HSI, hyperspectral imaging; FLIm, fluorescence lifetime imaging; OCT, optical coherence tomography; DOCI, dynamic optical contrast imaging; ^99m^Tc-MIBI, ^99m^Tc-sestamibi; SPECT/CT, single-photon emission computed tomography/computed tomography; PET/CT, positron emission tomography/computed tomography; MRI, magnetic resonance imaging; CT, computed tomography..

Rapid intraoperative PTH assay is a highly reliable and commonly used supplementary technique for identifying parathyroid glands, particularly for confirming that excised tissue is parathyroid in origin. One approach is to perform an intraoperative PTH measurement 10–20 minutes after total thyroidectomy (TT) and use an intact PTH (iPTH) plasma level <10 ng/L as a criterion for PAT, which is associated with a reduced incidence of transient hypocalcemia and a decreased risk of permanent hypoparathyroidism (7). Electrochemiluminescence immunoassay (ECLIA) has been used to measure PTH levels in fine-needle aspiration (FNA) samples, distinguishing parathyroid tissue (mean PTH 3369 pg/mL) from non-parathyroid tissue (25.7 pg/mL) (8). An innovative immunochromatographic test strip approach was developed to detect tissue PTH using the immunological colloidal gold technique (ICGT), demonstrating a substantially higher diagnostic accuracy for distinguishing parathyroid from non-parathyroid tissues compared with direct visual inspection (6 minutes for FNA and 2 minutes for tissue block homogenate [TBH]) (9).

Furthermore, near-infrared autofluorescence (NIRAF) enables parathyroid visualization without contrast when illuminated at a wavelength of approximately 800 nanometers (nm), with the parathyroid emitting fluorescence at approximately 820 nm due to internal fluorescent groups (10, 11). To further assess parathyroid vascular status and determine gland viability and functionality, exogenous fluorescent contrast agents such as indocyanine green (ICG) may be used. ICG is a cyanine dye used in medical diagnostics that rapidly binds to plasma lipoproteins after intravenous injection and emits fluorescence when activated by NIR light at approximately 800–820 nm, with a detection rate of 84%–100% (12, 13). Unexposed parathyroids can be mapped by NIRAF imaging at a maximum depth of 3.05 mm and an average depth of 1.23 mm, while ICG angiography can localize parathyroids within 3 mm of the adjacent tissue during the early stages of the operation (14). However, ICG may cause allergies and interfere with NIRAF, prompting the development of label-free non-invasive techniques such as laser speckle contrast imaging (LSCI), which monitors blood flow perfusion with 91.5% accuracy (15). Combining NIRAF and LSCI to create a parathyroid speckle and autofluorescence imager (ParaSPAI) has demonstrated that ParaSPAI can detect glands and assess their blood flow without compromising human health (16).

Alternative techniques for detecting parathyroid glands include bioelectrical impedance spectroscopy (BIS), which assesses the voltage response of biological tissues across several frequency ranges to derive the frequency spectrum of tissue impedance due to cellular structure and composition (17). Research indicates that BIS is a potentially effective tool capable of rapidly differentiating parathyroid tissue from adjacent tissues during surgery (18, 19). Additionally, other optical methods, including hyperspectral imaging (HSI), fluorescence lifetime imaging (FLIm), optical coherence tomography (OCT) imaging, and dynamic optical contrast imaging (DOCI), require further validation (20–23).

## 4 Whether to conduct PAT and the quantity of autotransplanted parathyroid tissue

**Figure 2:**
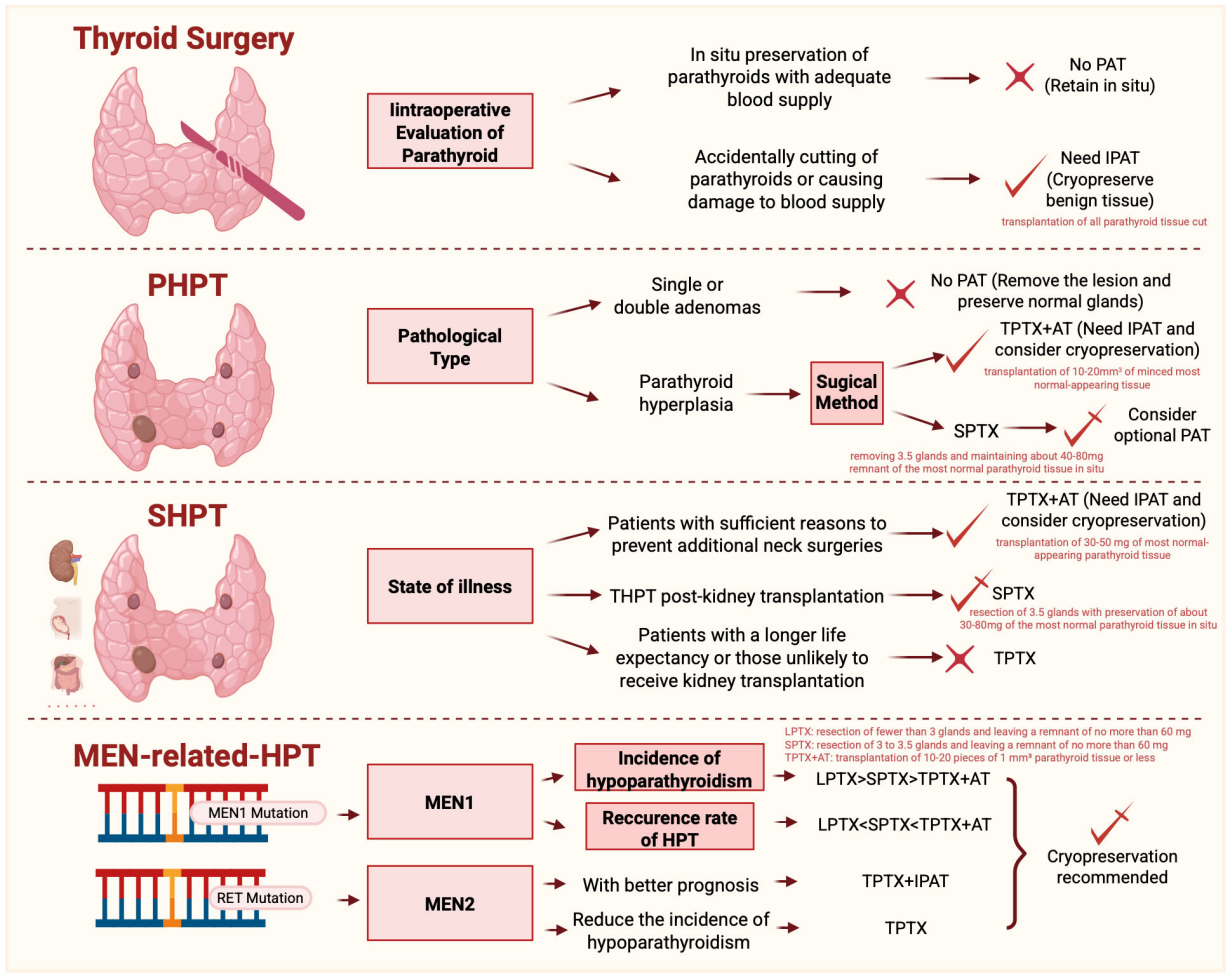
Decision flowchart for PAT in thyroid surgery, PHPT, SHPT, and MEN-related-HPT: whether to transplant and how much tissue to use. PAT, parathyroid autotransplantation; PHPT, primary hyperparathyroidism; TPTX, total parathyroidectomy; IPAT, immediate parathyroid autotransplantation; SPTX, subtotal parathyroidectomy; SHPT, secondary hyperparathyroidism; THPT, tertiary hyperparathyroidism; TPTX+AT, total parathyroidectomy with autotransplantation; MEN, multiple endocrine neoplasia; HPT, hyperparathyroidism; MEN1, multiple endocrine neoplasia type 1; MEN2, multiple endocrine neoplasia type 2; LPTX, less-than-subtotal parathyroidectomy.

Key factors influencing postoperative hypoparathyroidism include whether to perform PAT and the quantity of autotransplanted parathyroids. This decision-making process is not uniform but relies heavily on the specific indication for surgery, pathology, intraoperative findings, and patient-specific factors. The surgeon should strike a balance in maximizing autograft function to prevent permanent hypoparathyroidism while optimizing the quantity of autotransplanted tissue to minimize the risk of graft-dependent recurrence. PAT decisions across different clinical scenarios can be tailored as detailed below, including thyroidectomy, PHPT, SHPT, and MEN (**Figure 2**).

For patients undergoing initial thyroidectomy, research has found no association between PTH levels and the amount of autotransplanted parathyroids over a follow-up period of 6 months or longer. Moreover, the incidence of postoperative hypoparathyroidism was positively correlated with the number of autotransplanted parathyroid glands (24, 25). PAT was also identified as a risk factor for transient hypoparathyroidism (26). Regarding the impact of PAT on permanent hypoparathyroidism post-thyroidectomy, clinical research at different scales has produced conflicting conclusions. To clarify, a previous meta-analysis found the relationship unclear, whereas a more recent meta-analysis that included additional studies showed that PAT does not reduce the rate of permanent hypoparathyroidism compared with *in situ* preservation (25, 27). Autotransplanted parathyroid tissue does not recover function immediately; instead, the function generally begins to return approximately 4 weeks after surgery and progressively normalizes thereafter (28). PAT should not be performed routinely as a prophylactic measure, as it may needlessly increase the incidence of transient hypoparathyroidism without offering superior long-term protection compared with a viable *in situ* gland. Instead, PAT is best used as a selective rescue maneuver when the blood supply to the parathyroid gland is compromised, aligning with the 2025 American Thyroid Association (ATA) guidelines, which advocate preservation first (29).

In PHPT, the decision depends on the pathology and the extent of parathyroidectomy. Pathologically, 75%–80% of PHPT patients present with a single parathyroid adenoma, 15%–20% present with parathyroid hyperplasia, and parathyroid cancer is seldom seen (30). For single or double adenomas, PAT is unnecessary, as the hyperfunctioning gland can be surgically removed while normal glands are preserved. For hyperplasia managed with subtotal parathyroidectomy (SPTX), after removing 3.5 glands and maintaining a 40–80mg remnant of the most normal parathyroid tissue *in situ*, PAT may be performed or omitted based on postoperative PTH levels. Total parathyroidectomy (TPTX) requires urgent autotransplantation of 10–20mm^3^ of minced, normal-appearing tissue to avoid lifelong hypocalcemia (31, 32).

In cases of SHPT, SPTX, total parathyroidectomy with autotransplantation (TPTX+AT), and TPTX alone represent three contentious surgical interventions, with varying preferences across different countries and regions (33). Research indicates that TPTX+AT is the favored surgical method for patients with compelling reasons to avoid additional neck surgeries and for children and neonates, owing to its long-term efficacy in managing PTH levels, while SPTX is preferred for tertiary hyperparathyroidism (THPT) post-kidney transplantation because the incidence of recurrent SHPT is low (34–36). However, meta-analyses have shown no significant differences in symptom improvement, recurrence, or reoperation rates between TPTX+AT and SPTX (37, 38). In addition, a randomized controlled trial showed that treatment efficacy and complication rates were comparable among the three approaches (39). TPTX alone may be optimal for patients with a low likelihood of kidney transplantation or for elderly patients, as it shows no difference in surgical complication incidence or HPT persistence compared to TPTX+AT, while increasing the risk of hypoparathyroidism; however, it is more effective in reducing the likelihood of SHPT recurrence and a second surgery (40–42). For patients undergoing SPTX, resection of 3.5 glands, with preservation of approximately 30–80mg of the most normal parathyroid tissue *in situ*, is recommended (43, 44). For patients undergoing TPTX+AT, a study demonstrated that transplantation of 30–50 mg of parathyroid tissue is feasible, safe, and effective, with no transplant-dependent recurrence observed (45).

Multiple endocrine neoplasia type 1 (MEN1) and multiple endocrine neoplasia type 2 (MEN2) are relatively common genetic diseases that cause HPT, for which personalized surgery is crucial. In MEN1-related HPT, the choice among less-than-subtotal parathyroidectomy (LPTX), SPTX, and TPTX+AT balances the risk of hypoparathyroidism against HPT recurrence, with the incidence of hypoparathyroidism decreasing from LPTX to SPTX to TPTX+AT, mirroring the HPT recurrence rates (46, 47). Specifically, LPTX involves resection of fewer than three glands, whereas SPTX involves resection of 3–3.5 glands, leaving a remnant of no more than 60 mg in the neck. TPTX+AT entails resection of all parathyroid glands with transplantation of 10–20 pieces of 1 mm³ parathyroid tissue (47, 48). Moreover, Lambert et al. recommended using less PAT tissue than is typically used for sporadic PHPT, as this approach may reduce recurrence (49). Patients with MEN2 almost invariably harbor RET gene mutations and are at high risk of developing medullary thyroid carcinoma (MTC). Routinely performed prophylactic TPTX+AT demonstrates favorable prognostic outcomes, whereas *in situ* preservation of parathyroids during prophylactic TPTX may reduce the risk of hypoparathyroidism and represents a safe alternative approach (50, 51).

Patient characteristics that increase the risk of hypoparathyroidism or necessitate PAT as a therapeutic strategy should also be considered. A study found that among patients undergoing thyroidectomy, female sex, cN stage, gross extrathyroidal extension, (bilateral) central lymph node dissection, lateral lymph node dissection, TT, incidental parathyroidectomy (PTX), cancer, and node metastasis were associated with an increased risk of permanent hypoparathyroidism (26). Additional factors, including hypomagnesemia, preoperative vitamin D deficiency, thyroiditis, substernal multinodular goiters, and modified radical neck dissection, are associated with post-thyroidectomy hypocalcemia (52). Among patients with PHPT, younger age, elevated preoperative alkaline phosphatase levels, higher PTH levels, lower calcium levels, greater weight and number of resected parathyroid glands, an intraoperative PTH drop, and radiological changes in bone are associated with an increased risk of hypocalcemia post-PTX (53). Patients with SHPT who have higher preoperative serum calcium levels and lower iPTH levels are less likely to develop hypocalcemia after PTX (54). Moreover, research has found no significant associations between preoperative PTH, calcium, or phosphorus levels and recurrent HPT or permanent hypoparathyroidism in MEN1-related HPT, emphasizing the need for personalized surgical strategies (47). Integrating awareness of these risk profiles into the PAT decision allows surgeons to proactively identify high-risk patients and mitigate the incidence of complications.

## 5 PAT timing

Immediate parathyroid autotransplantation (IPAT) is an intraoperative procedure in which fresh parathyroid tissue is surgically transferred and implanted at an alternative site during surgery when there is a risk of devascularization or inadvertent removal, with the goal of preventing permanent hypoparathyroidism. IPAT exhibits high success rates, although reported figures vary considerably depending on how PAT success is defined. In earlier studies, when success was defined clinically as the ability to maintain a normal calcium level without calcium or vitamin D supplementation, IPAT success rates approached 100% (28, 55). In recent studies, in which IPAT success was defined biochemically by a PTH gradient >1.5 between the transplanted and non−transplanted arms, a stricter criterion for isolated graft function, reported success rates were approximately 85% (56, 57). This discrepancy arises because the clinical calcium level after thyroidectomy often reflects the combined function of both autografted and preserved *in situ* glands, whereas a PTH gradient specifically assesses graft−derived secretion. Current IPAT success rates have not reached 100%, mainly due to early graft ischemia, delayed revascularization (requiring 10–20 days to begin), and negative feedback suppression by preserved *in situ* parathyroid glands (57). To improve outcomes, research suggests immersing the parathyroid tissue in a nutritional solution for cell cultivation for 5 min before IPAT (58).

Cryopreserved parathyroid autotransplantation (CPAT) is a delayed autotransplantation method in which parathyroid tissue is cryopreserved during initial surgery for future reimplantation. It is primarily used in high-risk patients with permanent postsurgical hypoparathyroidism. However, the utilization rate of cryopreserved tissue is low, and reported CPAT success rates vary. Borot et al. and Shepet et al. both reported relatively low success rates. The former reported only 2 fully successful transplantations out of 22 patients who underwent CPAT, whereas the latter reported that 3 of 4 patients remained with hypoparathyroidism, with the sole successful case having undergone reimplantation only 4 days after the initial surgery (59, 60). In contrast, Schneider et al. found that among 15 CPAT patients (out of 883 patients with RHPT), 14 recovered normal PTH and calcium levels without necrosis, corresponding to a success rate approaching 100% (61). This significant discrepancy is largely attributable to quality control protocols and differences in institutional experience. Schneider et al. conducted histopathological assessments to ensure 0% necrosis and the absence of microbiological contamination in parathyroid tissue prior to reimplantation, a step that was not performed in the other two studies. Moreover, they used a standardized freezing and thawing protocol, and the interval between resection and freezing was always less than 1 h because the laboratory was located close to the operating suite. In a multicenter study such as that conducted by Borot et al., this level of standardization is more difficult to achieve. This underscores a critical determinant of CPAT success: without strict adherence to standardized quality control protocols, including pre-cryopreservation histopathological assessment to confirm tissue viability and absence of contamination, and minimization of ischemia time, the utility of CPAT remains severely limited.

Currently, there is no specific application scope for IPAT and CPAT in parathyroid tissue; however, they have different indications. IPAT is the preferred option for thyroidectomy, achieving high survival rates and minimizing permanent hypoparathyroidism (~1%) (62). The 2025 ATA guidelines suggest that devascularized or removed parathyroids should be autotransplanted into nearby muscle after analysis of a frozen section (of a tissue sample) confirms benign tissue (29). For PHPT caused by parathyroid hyperplasia, either IPAT or CPAT may be considered for SPTX, while IPAT is necessary for TPTX. A dual insurance strategy of IPAT+CPAT is recommended for recurrent non-hyperplasia PHPT due to uncertainty about residual gland adequacy. In SHPT, especially renal hyperparathyroidism (RHPT), survey data showed 40.7% of surgeons routinely perform IPAT, whereas 27.4% regularly perform cryopreservation, although some avoid it given low delayed autotransplantation needs (1%) and poor post-cryopreservation success rates (29, 63). In patients with THPT after renal transplantation, along with those with a prolonged life expectancy or those for whom renal transplantation is almost unfeasible, IPAT is redundant and CPAT is often not needed. IPAT is recommended for patients who have sufficient reasons to avoid repeat neck surgery, with CPAT serving as a backup strategy. For MEN1, a previous study reported 35% gland function retention after TPTX with IPAT and 31% with CPAT; recent data show 72% IPAT success versus 50% for CPAT (64, 65). Although success rates do not differ significantly, CPAT provides a salvage option if IPAT fails. For MEN2, due to the possibility of MTC patients undergoing multiple surgeries, irreversible parathyroid excision, or subsequent parathyroid malformation, cryopreservation during the initial surgery is recommended.

## 6 Processing autologous parathyroid

The fragmented transplantation technique preserves the structural integrity of tissue and, through precise cutting, facilitates rapid angiogenesis of the autotransplanted parathyroid tissue at the site of transplantation, thereby enhancing its viability. After meticulous excision of the adipose and fibrous tissue associated with the gland, the parathyroid is segmented into approximately 1–2mm³ fragments and dispersed into the muscle belly or subcutaneous tissue, which is subsequently sutured. This technique is usually employed in open surgery and CPAT; for instance, fragmented parathyroid tissue may be transplanted into the sternocleidomastoid muscle during thyroidectomy or thawed parathyroid tissue implanted into the brachioradialis muscle of the forearm. Non-absorbable sutures or metal clips aid possible future identification (66).

Comparatively, the tissue suspension injection technique is simpler to execute and causes less trauma. This method entails crushing the parathyroid glands in 1–2 mL of sterile physiological saline or balanced salt solution to create a suspension of small tissue clusters, which is then aspirated into a syringe. The suspension can be injected into multiple sites within the target muscle or subcutaneously via transdermal or direct needle insertion (67). This technique is frequently employed in both laparoscopic and open surgeries; for example, parathyroid tissue suspension may be injected into the deltoid muscle during transoral thyroidectomy or into the brachioradialis muscle of the forearm during open procedures.

Both techniques show comparable outcomes in thyroidectomy (68). However, it is noticeable that CPAT should employ the fragmented transplantation technique, as cryopreserved tissue is inherently more delicate, and preparing tissue suspension inflicts significant mechanical damage to cells. In contrast, tissue suspension increases contact between autografts and surrounding tissues while causing minimal damage to the parathyroid, thereby facilitating nutrient delivery and revascularization. Regarding reversibility, fragmented grafts are easier to locate during reoperation, making them more suitable for patients who may require secondary surgery, such as those with recurrent HPT requiring removal of subsequent lesions. In contrast, tissue suspension complicates graft localization due to its diffuse nature. Hence, selecting the appropriate technique based on the clinical context is of paramount importance.

While the techniques described above are highly effective, they may be limited by the risk of mechanical injury during processing and the finite supply of autologous tissue, especially in the event of permanent hypoparathyroidism after the first PAT. To address these limitations and enhance regenerative potential, innovative techniques such as the mesenchymal stem cell co-transplantation strategy have been developed. Research has shown that human embryonic stem cell lines (BG01-hESC and H1-hESC) and tonsil-derived mesenchymal stem cells (TMSCs) can differentiate into parathyroid-like cells that synthesize PTH (69–71). On this basis, Kim, H. Y., et al. injected differentiated TMSCs encapsulated in alginate microspheres (Al-dT) into rats post-PTX, resulting in a gradual elevation of iPTH levels (72). Co-injection with thermoreversible gels (e.g., PEG-PAF) as carrier materials for intramuscular PAT is also being explored (73). Although these advanced methods remain at the preclinical or early clinical stage, they may offer future salvage options for patients with non-functioning or non-viable parathyroid grafts.

## 7 PAT site

**Figure 3:**
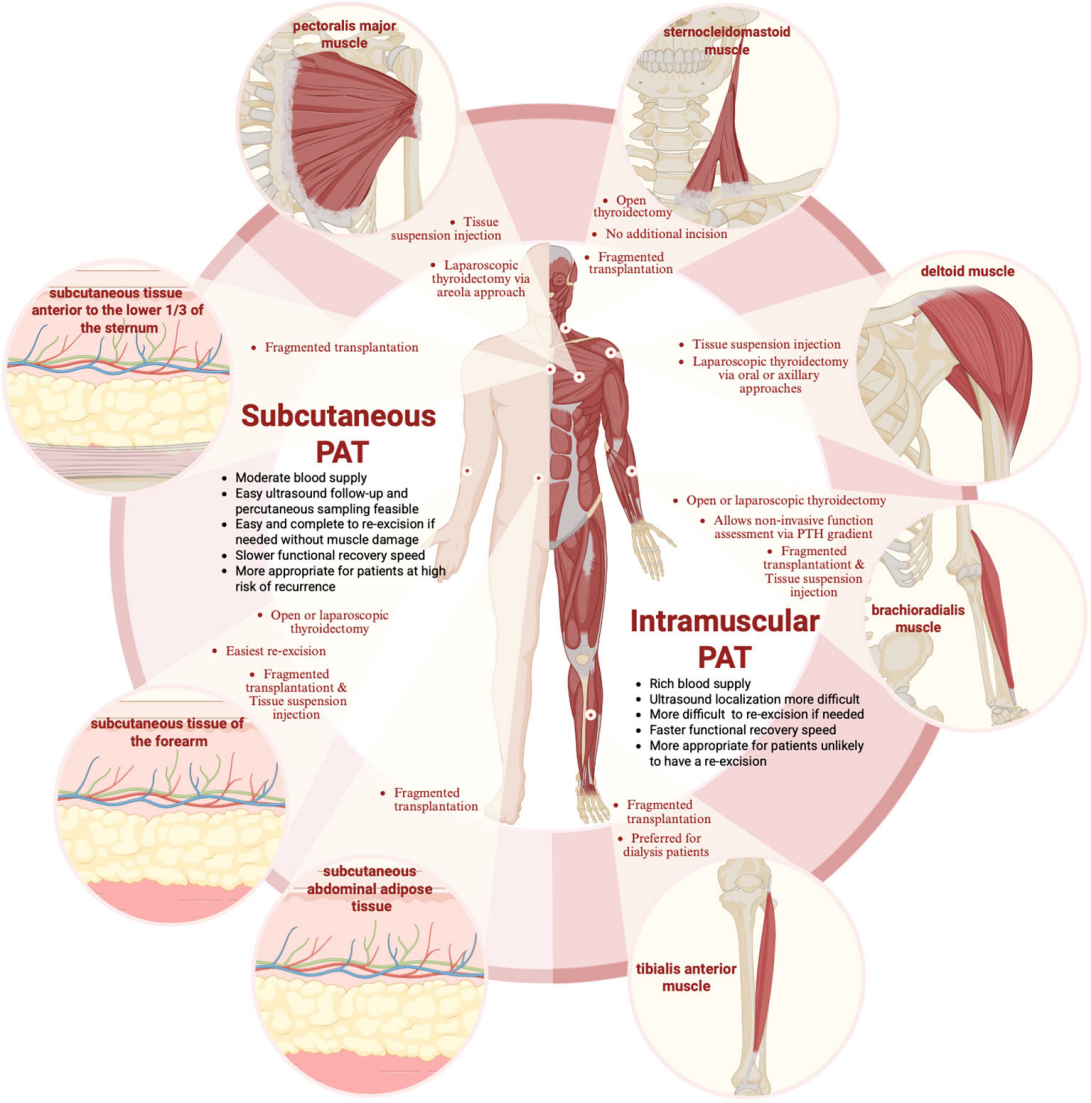
Site-specific considerations for PAT. PAT, parathyroid autotransplantation..

The ideal PAT site remains unstandardized and varies according to the clinical circumstance (**Figure 3**). The studied PAT sites include the sternocleidomastoid muscle, deltoid muscle, pectoralis major, forearm brachioradialis muscle, tibialis anterior, and the subcutaneous tissue of the forearm, sternum, and abdomen. These PAT locations endeavor to fulfill the optimal criteria for an autotransplantation site (1): ease of operation, safety, and minimal invasiveness (2); ample blood supply (3); reduced risk of complications; and (4) accessibility for on-site assessment of transplant functionality. It is important to note that the overall risk of site-specific complications, such as surgical infection, is very low because PAT is a clean (Class I) procedure using autologous tissue. Therefore, the choice among sites is driven less by differences in infection risk than by the balance between optimizing graft function and facilitating long-term management.

The sternocleidomastoid muscle has an ample blood supply and elevated oxygen levels, allowing its transplantation during surgery without an additional skin incision, making it the preferred PAT site for most thyroid surgeries (74). A limitation of sternocleidomastoid muscle PAT is its difficulty in reliably assessing the survival rate of parathyroid autografts, and if removal of the autografts is necessary, it heightens the likelihood of problems after a second cervical surgery.

The deltoid muscle is a feasible site for PAT because it is straightforward to access and does not interfere with the formation of vascular pathways in the event of reoperation. Intramuscular injection into the deltoid in THPT patients has been associated with a low recurrence rate (2.9%) (75). At present, deltoid PAT is frequently employed in endoscopic thyroidectomy via oral or axillary approaches, where parathyroid tissue is injected into the deltoid muscle.

Early pectoralis major PAT is often employed in open total thyroidectomy (TT), along with cervical lymph node dissection for differentiated thyroid carcinoma, showing PTH normalization within weeks (76, 77). Currently, pectoralis major PAT is usually utilized in endoscopic thyroidectomy via the areolar approach due to its rich vascular supply and the alignment of the surgical pathway through the chest area, where the pectoralis major is situated directly in the surgical route, eliminating the need for new puncture channels or added skin incisions for PAT.

The brachioradialis muscle of the forearm is a common surgical site in both open and laparoscopic thyroidectomy. It allows direct and sensitive assessment of autograft function by revealing the PTH gradient between the transplanted and non-transplanted arms. Lo CY et al. reported that reimplanted parathyroid tissue typically regained function within 2–4 weeks and fully recovered by 8 weeks (78). Abd Elmaksoud AE et al. observed successful autograft function in 25 of 26 patients with nasopharyngeal and laryngeal cancer who underwent prophylactic TT and forearm brachioradialis muscle PAT (79). Given that the majority of patients in this study were advanced cancer patients who had undergone preoperative radiotherapy or required postoperative radiotherapy, and considering that radical neck resection may involve excising the sternocleidomastoid muscle during initial or recurrent surgeries, forearm PAT is a superior option compared to neck PAT. A larger study (238 cases; 3-year follow-up) confirmed the safety and efficacy of forearm brachioradialis PAT, showing long-term survival rates of 85.7% for single transplants and 92.3% for double transplants (56). Moreover, Caliseo CT et al. found that single-site PAT at this location reduced surgical and anesthesia time compared with 20-site PAT in SHPT patients, without compromising graft function (80). Furthermore, the majority of CPAT transplant sites are located in the non-dominant brachioradialis muscle of the forearm, due to its ample blood supply and convenience for assessing autograft functionality (81).

The tibialis anterior is a favorable PAT site due to its surgical accessibility and resilience, allowing for extensive removal without compromising vital tissues during the invasive growth of autografts. Early research indicated that 30% of RHPT patients developed hypoparathyroidism following TPTX+AT surgery when the parathyroid was relocated to a site other than the tibialis anterior (82). Research by Anamaterou C et al. further supports its efficacy, demonstrating success rates of 86% (by PTH) and 96% (by calcium), with a 17% incidence of hypoparathyroidism in RHPT patients (83). For dialysis patients, the tibialis anterior is particularly advantageous as it preserves the forearm from potential complications related to arteriovenous fistula surgery and future vascular access needs.

Subcutaneous PAT places grafts in a fat-rich environment resembling the native physiological condition. Cavallaro G et al. demonstrated its efficacy in thyroid surgery, with 96% of patients regaining graft function by 3 months and 90.5% maintaining function at 1 year (84, 85). Since intramuscular implantation carries an increased risk—and up to 9% of recurrent SHPT cases ultimately require graft excision—subcutaneous forearm PAT represents a valuable alternative. Studies indicate that it provides safety and efficacy comparable to the intramuscular route, with similar rates of hypoparathyroidism (63). Subcutaneous forearm PAT may enhance parathyroid survival, facilitate evaluation in situations of recurrence requiring repeat surgery, and avoid functional impairment resulting from muscle resection.

The subcutaneous tissue anterior to the lower one-third of the sternum is another dependable PAT location. Postoperative outcomes for RHPT patients undergoing TPTX+AT at this location are comparable to those achieved with other sites, and hypertrophied grafts can be easily excised if recurrence occurs (86). A subsequent study demonstrated restoration of parathyroid function in RHPT patients undergoing CPAT by week 100, with a favorable recurrence rate of only 2.85%, affirming its long-term efficacy (87).

Subcutaneous abdominal adipose tissue can also aid in graft survival and function, allowing extensive resection, if required, without damaging critical structures. Jansson S et al. showed that parathyroid tissue can survive and operate within the abdominal subcutaneous adipose tissue in patients with PHPT and SHPT, with graft-dependent hypercalcemia resolving in two cases after autograft removal (88).

## 8 Evaluation of parathyroid autografts

Biochemical assessment of serum PTH and calcium levels is the core traditional method. Lo CY et al. first reported that postoperative hypocalcemia requiring medication to maintain normal blood calcium levels, together with low PTH levels one year after surgery, indicates permanent hypoparathyroidism (89). Subsequent studies underscored that authentic autograft function can only be validated by assessing PTH and blood calcium levels after discontinuation of calcium and vitamin D supplementation (90). Lorente Poch L et al. then proposed a more comprehensive definition of permanent transplant failure, whereby calcium medication, with or without calcitriol, remains necessary beyond one year post-transplantation and PTH levels are either subnormal (<13 pg/mL) or undetectable (91). The 2018 ATA guidelines suggest that postoperative PTH readings below 15 pg/mL often indicate a higher risk of acute hypoparathyroidism and that empirical or targeted oral calcium and vitamin D supplementation may help prevent its development. Combined iPTH and calcium monitoring during or post-thyroidectomy can optimize patient management (92).

For intramuscular PAT in the forearm, the Casanova test provides a non-invasive means of assessing autograft function and guiding clinical decision-making. A post-ischemia iPTH drop of >46% (positive) indicates that PTH originates from autografts, while a decrease of <20% (negative) suggests a neck or mediastinal source; a decline of 20%–46% is considered inconclusive (93). To improve practicality and avoid discomfort from total ischemia, a modified approach that compares bilateral forearm venous PTH levels has been developed. A PTH gradient (transplanted/non-transplanted side) exceeding 1.5 suggests normal autograft function, as the PTH released by the parathyroid autograft post-survival will first enter the venous return system of the transplanted forearm, resulting in a greater PTH concentration in the venous circulation on that side compared to the contralateral side (78). An ongoing analysis suggests that transplantation near antecubital veins (e.g., cephalic, median cubital, or basilic veins) rather than at distal sites may improve PTH gradient accuracy due to higher local PTH levels (94).​ Additionally, Anamateru C et al. validated a modified Casanova test in the tibial anterior muscle PAT using short-term limb ischemia and timed blood sampling, confirming its simplicity and efficacy (83).

Moreover, imaging techniques can be employed to assess the functionality of parathyroid autografts, including ultrasound, computed tomography (CT), radionuclide imaging, and magnetic resonance imaging (MRI). Among them, a popular approach combines high-frequency color ultrasound, ^99^mTc-sestamibi (^99^mTc-MIBI) single-photon emission computed tomography/computed tomography (SPECT/CT) fusion imaging, and MRI (4). High-frequency ultrasound can visualize autografts as small nodules and display their vascularity, indicating viability. ^99m^Tc-MIBI SPECT/CT fusion imaging offers valuable information on the endocrine functional status of transplant cells by providing their energy metabolism levels, and plays an important role in tracing the origin of recurrent lesions. Both ^18^F-choline and ^11^C-choline positron emission tomography/computed tomography (PET/CT) target membrane phospholipid synthesis in active cells, showing focal tracer uptake at transplant sites. ^18^F-choline PET/CT, often used after inconclusive ^99m^Tc-MIBI SPECT/CT, achieves a 90.3% detection rate, improving identification of hyperfunctional grafts (95). ^11^C-choline PET/CT, although costly, offers high predictive value and is reserved for ambiguous cases from ^99m^Tc-MIBI SPECT/CT and ^18^F-choline PET/CT (96). MRI, as a reliable auxiliary diagnostic tool, reveals healthy grafts via T2WI equal signals or slightly higher signals, in addition to pronounced and uniform enhancement of T1WI+c after injection of contrast agents. CT provides limited functional information, but its high spatial resolution offers advantages for precise anatomical localization and preoperative planning. The integration of these imaging techniques with biochemical markers and the (modified) Casanova test facilitates a multi-parameter visual assessment of the survival status, functional activity, and anatomical localization of autografts, thereby enhancing the comprehensiveness, accuracy, and sensitivity of autograft evaluation (**Table 1**).

## 9 Summary

**Figure 4:**
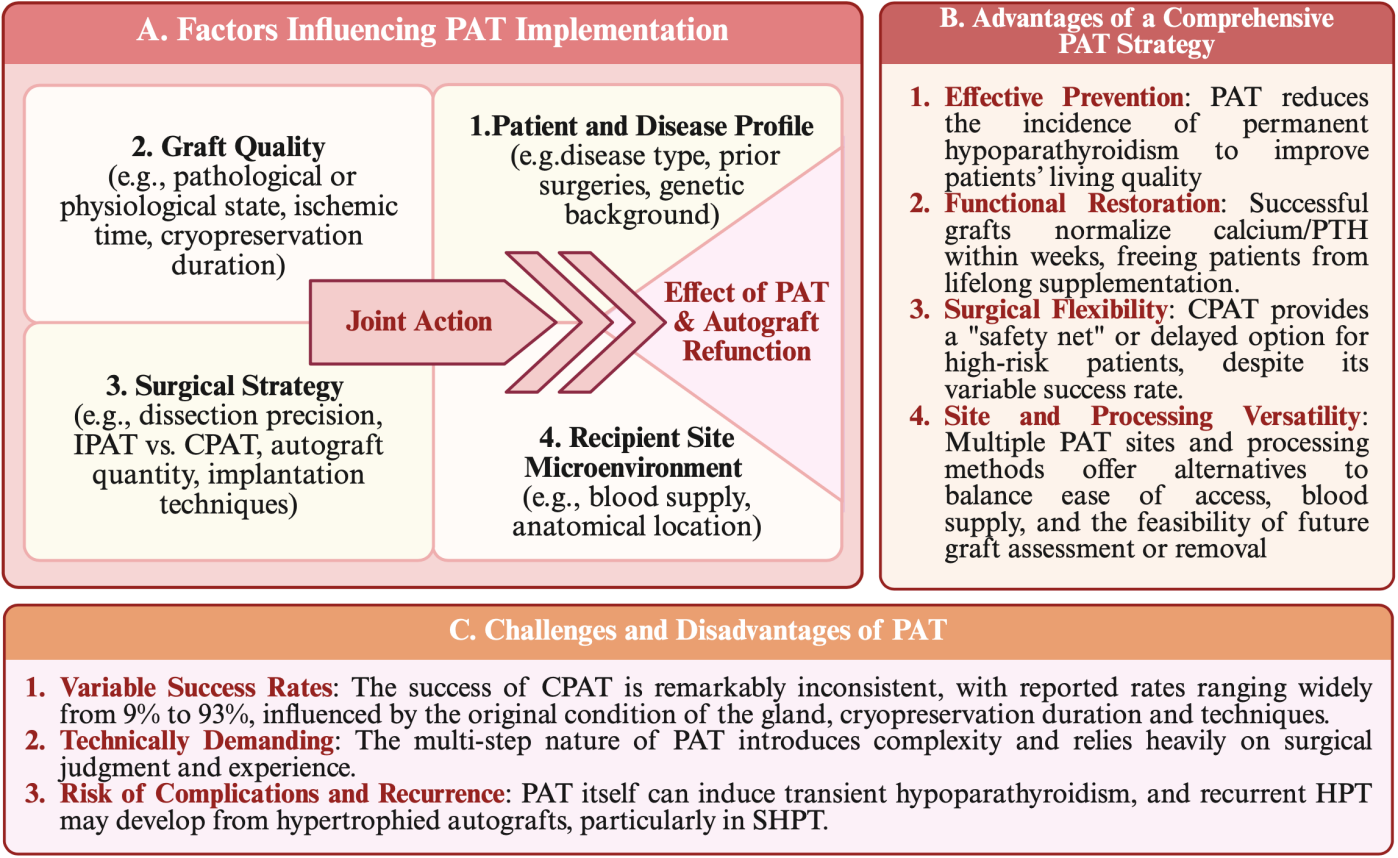
**(A)** Factors influencing PAT implementation; **(B)** Advantages of a comprehensive PAT strategy; **(C)** Challenges and disadvantages of PAT. PAT, parathyroid autotransplantation; IPAT, immediate parathyroid autotransplantation; CPAT, cryopreserved parathyroid autotransplantation; PTH, parathyroid hormone; SHPT, secondary hyperparathyroidism; HPT, hyperparathyroidism.

PAT is a pivotal surgical strategy for the prevention and treatment of permanent hypoparathyroidism following neck surgery. As this comprehensive review has shown, the procedure represents a dynamic continuum rather than a static technique, governed by the interaction among patient characteristics, graft quality, surgical strategy, and the recipient-site microenvironment (**Figure 4**). While the therapeutic value of PAT is well established, its current application is challenged by surgical heterogeneity. To optimize patient outcomes, future prospective research (particularly randomized trials) are needed to directly compare the efficacy of different autograft quantities across various situations, the infection, recurrence, complication rates, reversibility, cost-effectiveness. and quality-of-life outcomes associated with different PAT sites and processing techniques. Moreover, the wide variability in reported CPAT success rates highlights the need for standardized protocols and larger-scale studies. Emerging technologies also warrant further investigation to enhance diagnostic precision, improve long-term graft viability, and provide alternative solutions for patients with permanent hypoparathyroidism.

This study has several limitations. First, the restriction to English-language publications and the exclusion of very small case series may have resulted in the exclusion of relevant data. Second, the findings are subject to potential publication bias, as studies demonstrating successful autotransplantation are more likely to be published than those reporting graft failure, potentially inflating the perceived efficacy of PAT. Third, given heterogeneity in design and outcomes, evidence synthesis was largely narrative rather than quantitatively pooled; therefore, conclusions should be applied with clinical judgment and local expertise.

In conclusion, PAT emerges as a dynamic and adaptable surgical technique whose therapeutic potential can be maximized through careful consideration of the clinical context. While its multifaceted nature poses challenges to standardization, the consolidated evidence in this review underscores its indispensable role in preserving endocrine homeostasis and improving quality of life. This review provides an up-to-date synthesis of the current evidence and, through the introduction of a novel phase-based framework, offers a comprehensive overview of the PAT procedure and a structure-optimizing PAT.

## Glossary

PAT: parathyroid autotransplantation
PTH: parathyroid hormone
PHPT: primary hyperparathyroidism
SHPT: secondary hyperparathyroidism
HPT: hyperparathyroidism
MEN: multiple endocrine neoplasia
TT: total thyroidectomy
iPTH: intact parathyroid hormone
ECLIA: electrochemiluminescence immunoassay
FNA: fine-needle aspiration
ICGT: immunological colloidal gold technique
TBH: tissue block homogenate
NIRAF: near-infrared light autofluorescence
ICG: indocyanine green
LSCI: laser speckle contrast imaging
ParaSPAI: parathyroid speckle and autofluorescence imager
BIS: bioelectrical impedance spectroscopy
HSI: hyperspectral imaging
FLIm: fluorescence lifetime imaging
OCT: optical coherence tomography
DOCI: dynamic optical contrast imaging
ATA: American Thyroid Association
SPTX: subtotal parathyroidectomy
TPTX: total parathyroidectomy
TPTX+AT: total parathyroidectomy with autotransplantation
THPT: tertiary hyperparathyroidism
MEN1: multiple endocrine neoplasia type 1
MEN2: multiple endocrine neoplasia type 2
LPTX: less-than-subtotal parathyroidectomy
MTC: medullary thyroid carcinoma
PTX: parathyroidectomy
IPAT: immediate parathyroid autotransplantation
CPAT: cryopreserved parathyroid autotransplantation
RHPT: renal hyperparathyroidism
TMSCs: mesenchymal stem cells
Al-dT: differentiated TMSCs encapsulated in alginate microspheres
PEG-PAF: polyethylene glycol polyanine co-phenylalanine
CT: computed tomography
MRI: magnetic resonance imaging
99mTc-MIBI: 99mTc-sestamibi
SPECT/CT: single-photon emission computed tomography/computed tomography
PET/CT: positron emission tomography/computed tomography.
