# Supplementary material for: Parathyroid autotransplantation: a phase-based framework for surgical decision-making
Source: Front Endocrinol (Lausanne). 2026 Jul 9;17:1863354. doi: 10.3389/fendo.2026.1863354 (PMC13391414; doi:10.3389/fendo.2026.1863354)
Supplement: Supplementary file 1 [file DataSheet1.pdf]

**Table S1.** Detailed search strategy of our review.

| Concept | Search Strategy |
|---|---|
| Parathyroid | "Parathyroid Glands"[Mesh] OR "parathyroids" OR "parathyroid gland" OR "parathyroid glands" OR "parathyroid tissue" |
| Autotransplantation | "Transplantation, Autologous"[Mesh] OR "autotransplantation" OR "auto-transplantation" OR "autotransplant" OR "autotransplanted" OR "reimplantation" OR "re-implantation" OR "autograft" OR "autografts" OR "autologous" OR "autografting" |
| Identification and evaluation (intraoperative) | "Identif*" OR "locat*" OR "locali*" OR "detect*" OR "distinguish*" OR "visual*" OR "assess*" OR "evaluat*" OR "map*" OR "recogniz*" OR "specif*" OR "discover*" OR "reveal*" OR "discern*" OR "find*" OR "found" OR "judg*" OR "determin*" OR "verif*" OR "estimate*" OR "gauge" OR "anatom*" OR "test" OR "surgeon" OR "assay" OR "exam*" OR "trial" OR "PTH" OR "parathyroid hormone" OR "NIRAF" OR "autofluorescence" OR "bio*" OR "optical" OR "imag*" |
| Thyroid surgery | "Thyroidectomy"[Mesh] OR "thyroidectomies" OR "thyroid surgery" OR "neck dissection" OR "lymph node dissection" |
| Primary hyperparathyroidism | "Hyperparathyroidism, Primary"[Mesh] OR "hyperparathyroidism, primary" OR "primary hyperparathyroidism" OR "primary hyperparathyroidisms" OR "parathyroidectom*" |
| Secondary hyperparathyroidism | "Hyperparathyroidism, Secondary"[Mesh] OR "hyperparathyroidisms, secondary" OR "secondary hyperparathyroidism" OR "secondary hyperparathyroidisms" OR "parathyroidectom*" OR "hyperparathyroidisms, tertiary" OR "tertiary hyperparathyroidism" OR "tertiary hyperparathyroidisms" |
| Multiple endocrine neoplasia | "Multiple Endocrine Neoplasia"[Mesh] OR "multiple endocrine neoplasms" OR "neoplasms, multiple endocrine" OR "neoplasia, multiple endocrine" OR "endocrine neoplasia, multiple" OR "endocrine neoplasms, multiple" OR "adenomatosis, multiple endocrine" OR "adenomatoses, multiple endocrine" OR "endocrine adenomatosis, multiple" OR "endocrine adenomatoses, multiple" OR "multiple endocrine adenomatoses" OR "multiple endocrine adenomatosis" OR "multiple endocrine neoplasia syndromes" OR "multiple endocrine adenopathy" OR "multiple endocrine adenopathies" OR "adenopathy, multiple endocrine" OR "adenopathies, multiple endocrine" OR "endocrine adenopathy, multiple" OR "endocrine adenopathies, multiple" |
| Timeliness | "Immediate*" OR "instant*" OR "simultaneous*" OR "synchroniz*" OR "delay*" OR "metachronous*" OR "cryopreserv*" OR "IPAT" OR "CPAT" |
| Processing methods | "Process*" OR "manipulat*" OR "fragment*" OR "inject*" OR "suspension" OR "novel" OR "innovat*" OR "emerging" OR "new*" OR "alternat*" OR "modern" OR "updat*" OR "advance*" |
| Site | (Site) OR (locat*) OR (place) OR (position) OR (situat*) OR (sternocleidomastoid) OR (deltoid) OR (pectoralis) OR (brachioradialis) OR (foream) OR (tibialis) OR (muscle) OR (subcutaneous*) |
| Identification and evaluation (post-PAT) | "Identif*" OR "locat*" OR "locali*" OR "detect*" OR "distinguish*" OR "visual*" OR "assess*" OR "evaluat*" OR "map*" OR "recogniz*" OR "specif*" OR "discover*" OR "reveal*" OR "discern*" OR "find*" OR "found" OR "judg*" OR "determin*" OR "verif*" OR "estimate*" OR "gauge" OR "test" OR "assay" OR "exam*" OR "trial" OR "PTH" OR "parathyroid hormone" OR "bio*" OR "calcium" OR "vitamin D" OR "Casanova" OR "PTH gradient" OR "ultrasound" OR "computed tomography" OR "CT" OR "radionuclide imaging" OR "99mTc-sestamibi" OR "99mTc-MIBI" OR "18F-choline" OR "11C-choline" OR "magnetic resonance imaging" OR "MRI" |
